# NAMPT overexpression induces cancer stemness and defines a novel tumor signature for glioma prognosis

**DOI:** 10.18632/oncotarget.20577

**Published:** 2017-08-28

**Authors:** Antonio Lucena-Cacace, Daniel Otero-Albiol, Manuel P. Jiménez-García, Javier Peinado-Serrano, Amancio Carnero

**Affiliations:** ^1^ Instituto de Biomedicina de Sevilla, IBIS, Hospital Universitario Virgen del Rocio, Universidad de Sevilla, Consejo Superior de Investigaciones Cientificas, Sevilla, Spain; ^2^ CIBER DE CANCER, Instituto de Salud Carlos III, Madrid, Spain

**Keywords:** NAMPT, cancer initiating cell, gene signature, glioma, glioblastoma

## Abstract

Gliomas are the most prevalent primary malignant brain tumors associated with poor prognosis. NAMPT, a rate-limiting enzyme that boosts the nicotinamide adenine dinucleotide (NAD) regeneration in the salvage pathway, is commonly expressed in these tumors. NAD metabolism is required to maintain tissue homeostasis. To maintain metabolism, cancer cells require a stable NAD regeneration circuit. However, high levels of NAD confer resistance to therapy to these tumors, usually treated with Temozolomide (TMZ). We report that NAMPT overexpression in glioma cell lines increases tumorigenic properties controlling stem cell pathways and enriching the cancer-initiating cell (CIC) population. Furthermore, NAMPT expression correlated with high levels of Nanog, CD133 and CIC-like cells in glioblastoma directly extracted from patients. Meta-analysis reveals that NAMPT is also a key factor inducing cancer stem pathways in glioma cells. Furthermore, we report a novel NAMPT-driven signature which stratify prognosis within tumor staging. NAMPT signature also correlates directly with EGFR positive and IDH negative tumors. Finally, NAMPT inhibition increases sensitivity to apoptosis in both NAMPT-expressing cells and tumorspheres. Therefore, NAMPT represents a novel therapeutic target in Glioma progression and relapse.

## INTRODUCTION

Gliomas are the most lethal and prevalent primary brain tumors in adults and are associated with a poor median survival time, which barely exceeds 12 months despite newly available treatments [1, 2]. These unsuccessful attempts to manage gliomas have stimulated research for more effective therapies. Several studies have highlighted the importance of intratumoral heterogeneity, which is driven by genetic and epigenetic effectors, to therapeutic responses and patient survival, especially in gliomas. Tumor heterogeneity is partially explained by the cancer-initiating cell (CIC) hypothesis, which states that a cellular hierarchy exists in some tumors with self- renewing CICs, generating the progeny that are responsible for tumor complexity [3, 4]. CICs express certain stem cell markers and exhibit sustained self-renewal. CICs also display high radio- and chemoresistance, which contribute to tumor relapse following treatment [5–9]. Thus, targeting CICs offers a potential new treatment frontier for glioma control.

The recently elucidated concepts of metabolic reprogramming and oncogenic metabolites support the key role played by metabolism during the transformation of somatic cells to cancer cells [10]. Nutrient acquisition and utilization are critical for tumor progression, and these metabolic alterations are enhanced by the Warburg effect [11, 12]. Tumor cells become less reliant on oxygen-dependent mitochondrial oxidative phosphorylation and instead rely on alternative pathways to facilitate enzymatic activity, such as anaerobic glycolysis or the NAD salvage pathway. This metabolic reprogramming generates products that are required to produce building blocks, such as proteins, lipids and nucleic acids, to facilitate tumor progression. The brain is an extremely metabolically active organ that derives energy almost entirely from glucose but nonetheless requires extra sources of NAD to maintain an abnormal metabolic state [13, 14]. Interestingly, it has been found that in the brain, high levels of NAD protect against cell death in the absence of glucose [15]. Thus, NAD production may play a key role in brain tumor initiation, progression and relapse.

Beyond its role as a metabolite, NAD derivatives function as important cofactors in cellular redox reactions and as a second messenger in a large number of cellular processes. It exists in two forms, an oxidized (NAD+) and a reduced (NADH) form. NAD+ is essential for metabolism, energy production, DNA repair, and signaling in many types of cancer cells [13, 14]. NAD is synthesized from nicotinamide, nicotinic acid, or tryptophan. NAD is primarily synthesized from nicotinamide, a process known as the NAD salvage pathway. Nicotinamide phosphoribosyl transferase (NAMPT) catalyzes the conversion of nicotinamide to nicotinamide mononucleotide (NMN), which is the rate-limiting step in the NAD salvage pathway. NAMPT is essential for NAD biosynthesis. Inhibition of NAMPT leads to depletion of NAD+, which in turn inhibits ATP synthesis [16]. This effect eventually causes attenuation of cancer cell proliferation and death [17, 18]. NAMPT inhibition also leads to attenuation of glycolysis, resulting in further perturbation of carbohydrate metabolism in cancer cells [18, 19]. Therefore, NAMPT has been proposed as a promising anticancer target [20, 21].

NAMPT is upregulated in several human malignancies, including breast, colon, prostate, thyroid, gastric, and several hematopoietic malignancies. In some malignancies, such as sarcomas and gastric thyroid and prostate carcinomas, higher NAMPT expression correlates with deeper tumor invasion and increased metastatic potential and chemotherapy resistance [15, 22–24].

In this work, we systematically explored the role of NAMPT in gliomas and demonstrated that NAMPT is a strong oncogene that induces essential developmental and stem cell pathways, facilitating the dedifferentiation of tumor cells into CICs. Based on these results, we identified a gene signature triggered by NAMPT that correlates with tumor stage and poor prognosis. Furthermore, we showed that NAMPT represents a promising target for future glioma treatments focused on the CIC subpopulation.

## RESULTS

### NAMPT correlates with glioma tumor clinical outcomes

**Figure 1:**
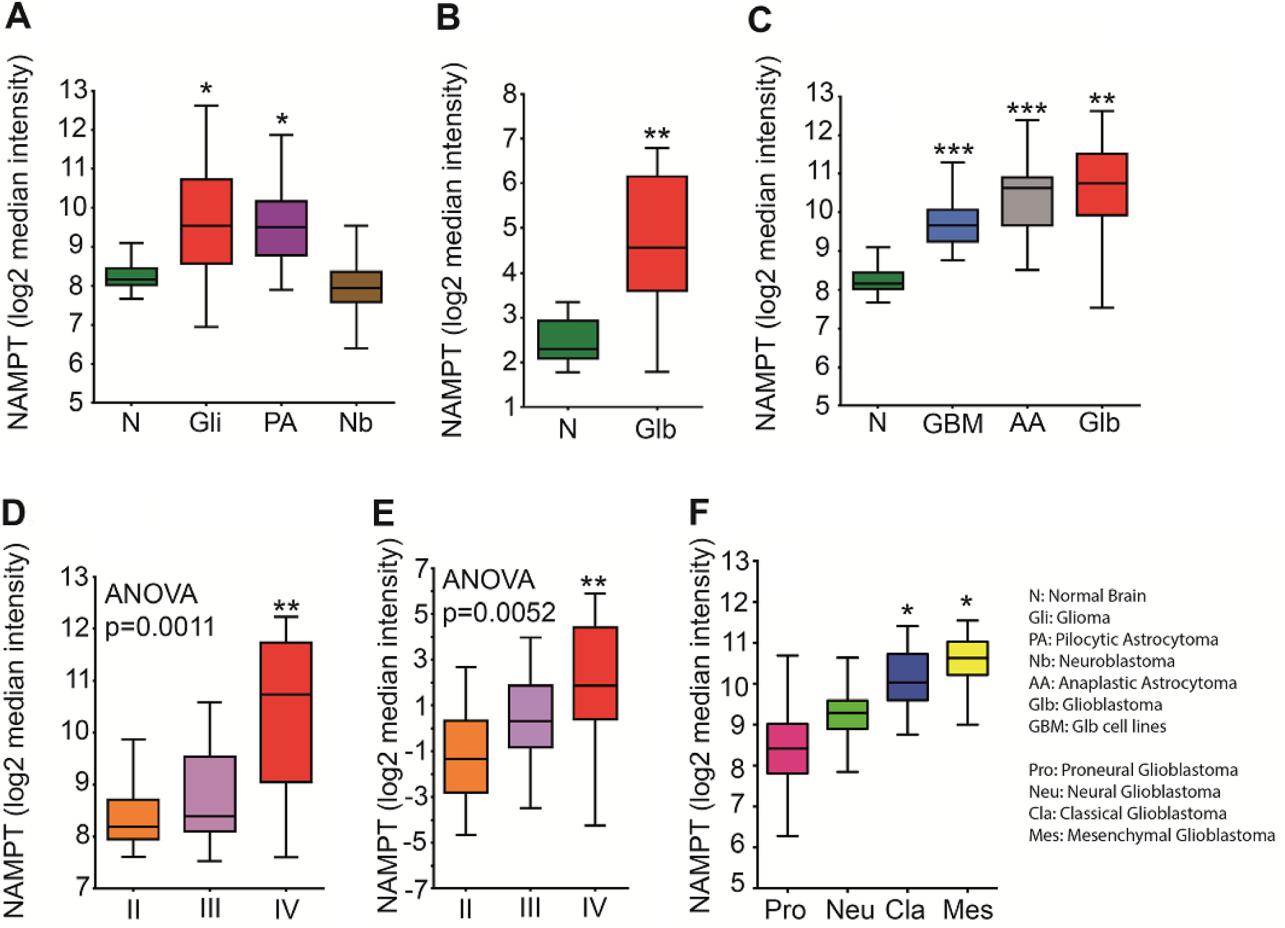
NAMPT expression correlates with tumor grade (**A**) Analysis of Normal Brain [*n =* 216], Glioma [*n =* 577], Pilocytic Astrocytoma [*n =* 41] and N euroblastoma [*n =* 45] datasets available through the R2 database indicates that NAMPT is significantly upregulated in tumors derived from the ectoderm, such as pilocytic astrocytoma [^*^p = 0.01 with ANOVA compared to normal brain] and glioma [^*^p = 0.01 with ANOVA compared to normal brain]. (**B**) Analysis of normal brain [*n =* 23] and glioblastoma [*n =* 81] datasets from the Sun database available in Oncomine indicates that NAMPT is highly overexpressed in glioblastoma [^**^p < 0.01 with ANOVA compared to normal brain]. (**C**) New retrospective analyses of new databases, comparing normal brain GSE13564, GSE11882 [*n =* 216], Glioblastoma [GSE4290, GSE16011 Glioblastoma series taken together; *n =* 159], Anaplastic Astrocytoma - glioma grade III [GSE4290, Astrocytoma grade III GSM series; *n =* 16] and Glioblastoma [*n =* 9; GSM379855, GSM379856, GSM379857, GSM379858, GSM379870, GSM379871, GSM379872, GSM379873 and GSM379874] cell lines shows that NAMPT is upregulated in the mRNA pools of these cell lines [GSE15209 ^****^p < 0.0001 with ANOVA compared to normal brain] and that its expression is upregulated at baseline in glioma grade III cells [^***^p < 0.001 with ANOVA compared to normal brain]. (**D**–**E**) The Oncomine glioma dataset shows a correlation between NAMPT expression and tumor grade. (D) Analysis of the Oncomine Glioma dataset stratifying NAMPT according to Glioma grade with Grade II [*n =* 45] Grade III [*n =* 31] and grade IV [*n =* 81] tumors shows a significant difference in NAMPT expression between grade II and grade IV tumors [^**^p < 0.001]. (E) A second database analyzed in Oncomine strengthens the data previously shown: NAMPT expression correlating with tumor grade: Grade II [*n =* 50], Grade III [*n =* 26], Grade IV [*n =* 77] shows a significant difference in NAMPT expression between grade II and grade IV tumors [^**^p = 0.0052] (**F**) Analysis of TCGA Glioblastoma molecular subtypes Proneural, Neural, Classical and Mesenchymal.

To confirm the clinical significance of NAMPT expression in brain tumors, we analyzed public available ectoderm-derived tumor datasets for NAMPT levels (Figure 1A). We determined that gliomas, pilocytic astrocytomas but not in neuroblastomas express more NAMPT than healthy human brain tissue. We found that NAMPT was highly overexpressed in glioblastoma tissue compared with healthy brain tissue (Figure 1B). This correlation was also observed in an mRNA pool of nine different glioma cell lines (Figure 1C), as well as in astrocytoma, glioma grade III, and glioblastoma, glioma grade IV tumors (Figure 1C). Furthermore, NAMPT expression was strongly correlated with tumor grade in two independent datasets (Figure 1D–1E), indicating that NAMPT is highly overexpressed in human brain tumors and correlates with tumor stage. According to the TCGA molecular classification of Glioblastoma, we found that NAMPT is particularly expressed in the Classical and Mesenchymal subtype. (Figure 1F)

**Figure 2:**
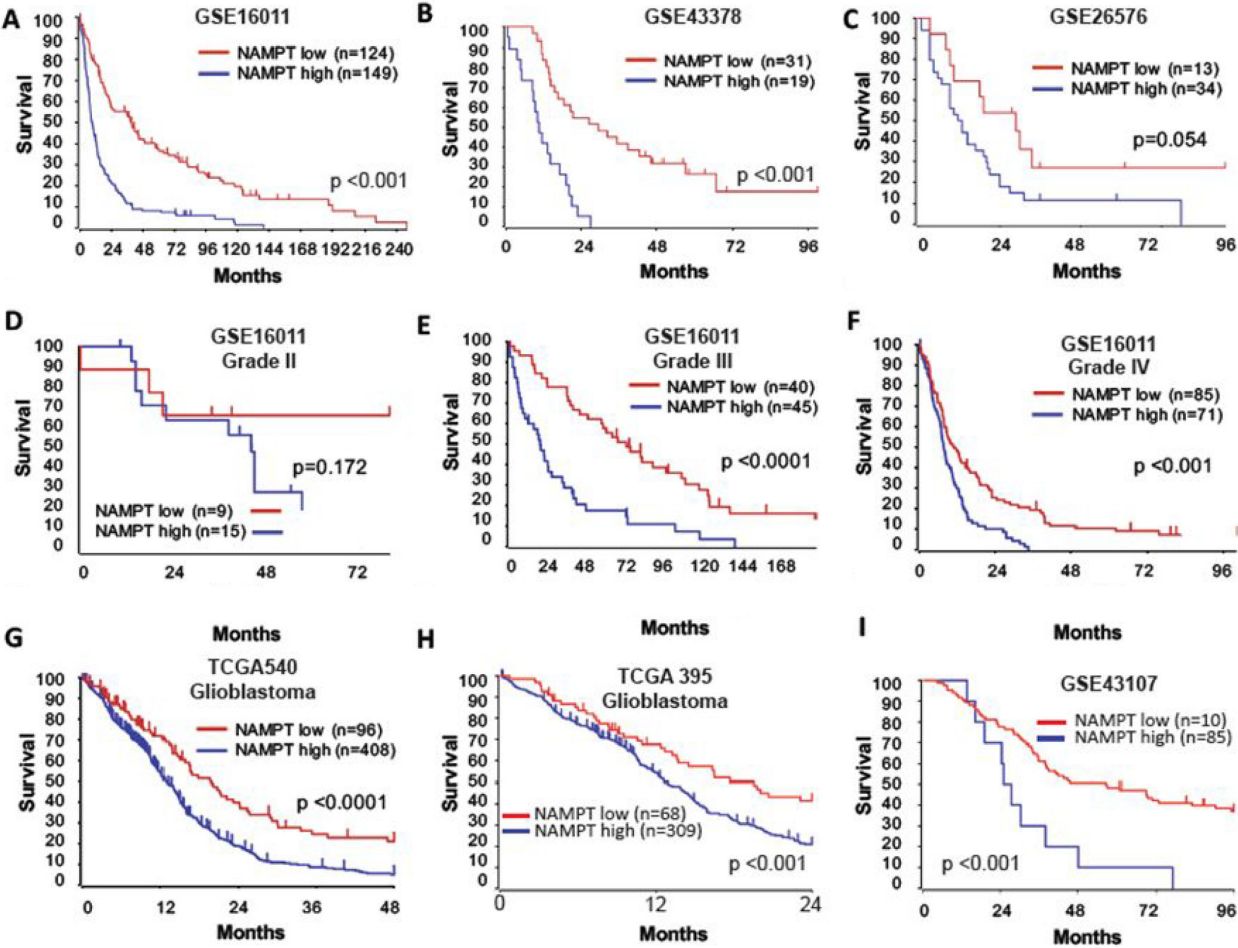
NAMPT is an independent indicator of glioma patient outcomes Analysis of glioma datasets available through Oncomine and R2 indicates the existence of a significant correlation between high NAMPT expression and poor survival in the French tumor glioma dataset (**A**) [*n =* 124 NAMPT low; *n =* 149 NAMPT high; *p =* 3,20E- 13 with log-rank analysis]; Kawaguchi tumor glioma dataset. (**B**) [*n =* 31 NAMPT low; *n =* 19 NAMPT high; *p =* 1.3E-05 with log-rank analysis]; Paugh tumor glioma dataset. (**C**) [*n =* 13 NAMPT low; *n =* 34 NAMPT high; *p =* 0.054 with log-rank analysis]; French tumor glioma dataset, subtype grade II. (**D**) [*n =* 9 NAMPT low; *n =* 15 NAMPT high; *p =* 0.172 with log-rank analysis]; French tumor glioma dataset, subtype grade III. (**E**) [*n =* 40 NAMPT low; *n =* 45 NAMPT high; *p =* 0.000018 with log-rank analysis]; and French tumor glioma dataset, subtype grade IV. (**F**) [*n =* 85 NAMPT low; *n =* 71 NAMPT high; *p =* 0.000360 with log-rank analysis]. The poor prognosis of grade IV glioblastoma is confirmed via analysis of the TCGA 540 glioblastoma dataset [*n =* 96 NAMPT low; *n =* 408 NAMPT high; *p =* 0.000071 with log-rank analysis] (**G**), TCGA 395 glioblastoma dataset [*n =* 68 NAMPT low; *n =* 309 NAMPT high; *p =* 0.00089 with log-rank analysis] (**H**) and French Exon-Core dataset [*n =* 85 NAMPT low; *n =* 10 NAMPT high; *p =* 0.0026 with log-rank analysis] (**I**).

To further evaluate the potential correlation between NAMPT expression and patient outcome, we generated Kaplan-Meier survival curves from several public databases (Figure 2). In all datasets analyzed, high NAMPT expression was indicative of poor survival (Figure 2A–2C). Because NAMPT is closely associated with tumor stage, we segregated patients by tumor grade to determine whether the effects of the enzyme on survival were stage-related or stage-independent. NAMPT correlated with poor prognosis independently of tumor grade in all datasets (Figure 2D–2F). We also found that glioblastoma grade IV tumors expressing high levels of NAMPT had a worse prognosis (Figure 2G), which was confirmed in other datasets (Figure 2H–2I).

Altogether, these data demonstrate that NAMPT expression in gliomas is an independent indicator of poor patient outcomes, which may indicate that NAMPT has an important oncogenic function in glioma cells -.

### NAMPT strengthens tumorigenic properties enriching cancer initiating cell phenotype

**Figure 3:**
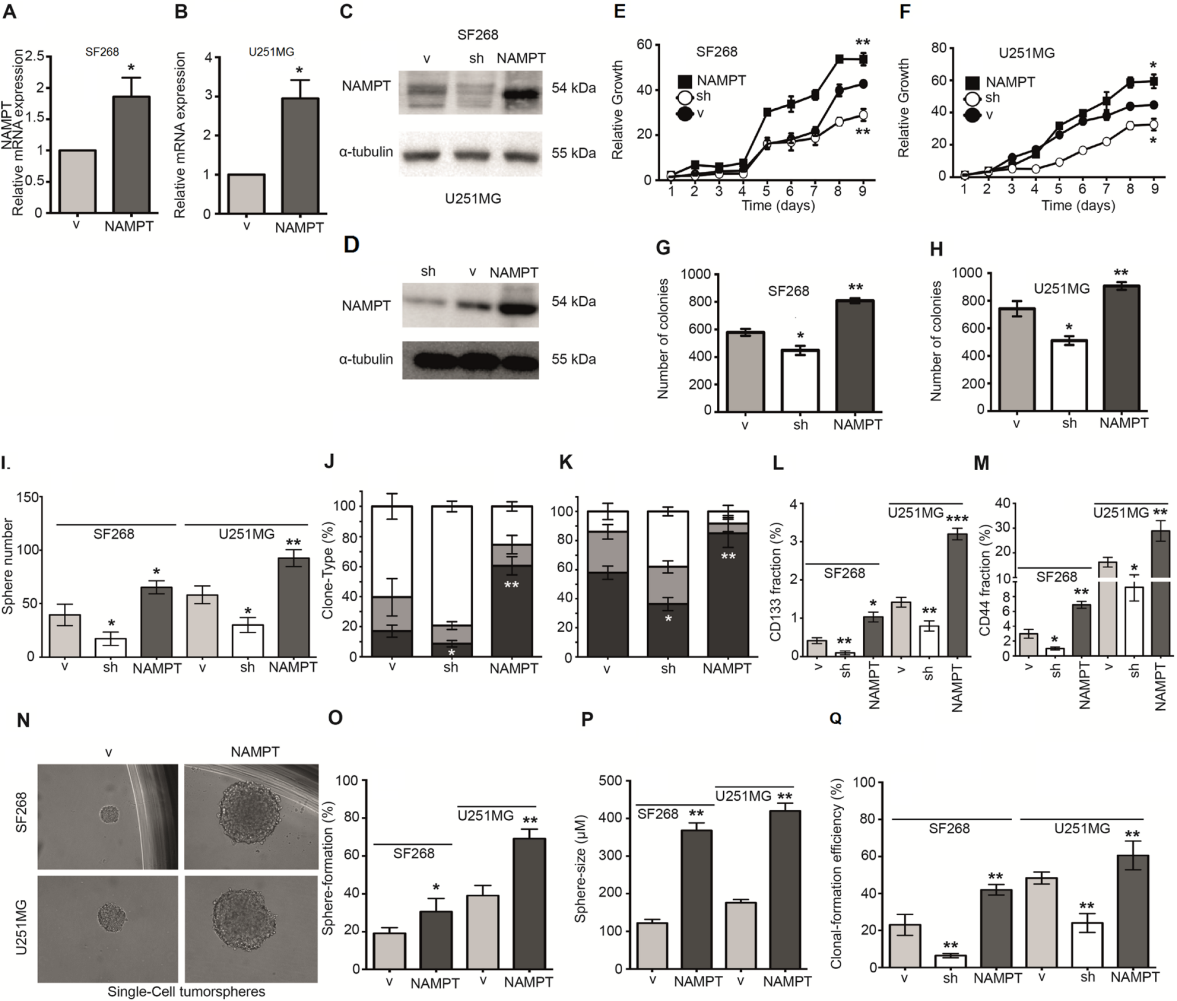
NAMPT expression increases tumorigenic and CIC properties (**A**, **B**) Q-RT-PCR shows NAMPT overexpression in either SF268 (A) or U251MG (B). (C, F) Western blot analysis shows NAMPT overexpression and NAMPT silencing with shRNA in SF268 (**C**) and U251MG (**D**). (**E**, **F**) Analysis of the growth curve indicates that NAMPT overexpression confers a proliferative advantage [^*^p < 0.05; ^**^p < 0.01 with ANOVA compared to vector], whereas NAMPT underexpression slows proliferation [^*^p < 0.05, ^**^p < 0.01 with ANOVA compared to vector]. (**G**, **H**) Clonogenicity assay results indicate that NAMPT overexpression increases the number of colonies [^**^p < 0.01 with ANOVA compared to vector], whereas NAMPT underexpression decreases the number of colonies [^*^p < 0.05 with ANOVA compared to vector]. (**I**) Tumorsphere-forming assay in both cell lines indicates that NAMPT overexpression increases both number and size, whereas NAMPT underexpression decreases tumorsphere number and size. (**J**, **K**) Analysis of clone phenotypes [holoclones – black, meroclones – gray, paraclones – white] shows that NAMPT overexpression increases the number of holoclones [^**^p < 0.01 with ANOVA compared to vector], whereas NAMPT underexpression decreases the holoclone number [^*^p < 0.05 with ANOVA compared to vector]. J shows data from SF268 cell line. K shows data from U251MG cell line. (**L**) CD133 analysis with FACS indicates that NAMPT overexpression increases CD133 levels [^*^p < 0.05 with ANOVA compared to vector], whereas NAMPT underexpression decreases CD133 levels [^**^p < 0.01 with ANOVA compared to vector]. (**M**) CD44 analysis with FACS indicates that NAMPT overexpression increases CD44 levels [^**^p < 0.01 with ANOVA compared to vector], whereas NAMPT underexpression decreases CD44 levels [^*^p < 0.05 with ANOVA compared to vector]. (**N**–**Q**) Single-cell Sphere-forming assay indicates that NAMPT overexpression increases both number and size, whereas NAMPT underexpression decreases tumorsphere number and size. (N) Single cell tumorsphere forming efficiency representative picture. (O) Single cell tumorsphere forming efficiency percentage. (Q) Single cell tumorsphere size. (P) Single cell colony (full culture) forming efficiency percentage.

To elucidate the causal role of NAMPT, we studied whether NAMPT promotes tumorigenicity in glioma cells. We ectopically expressed NAMPT cDNA (Isoform A) in the human glioblastoma cell lines SF268 and U251MG, then selected transfectants to create a stable transfection pool (NAMPT) (Figure 3A–3D). As proof of concept, we also counteracted endogenous NAMPT gene expression by expressing a small hairpin RNA against NAMPT (sh), producing a daughter cell line expressing reduced levels of the enzyme (Figure 3C–3D). We then compared the behavior of the parental cell line (expressing only vector, V) with that of isogenic daughter cells exhibiting NAMPT overexpression (NAMPT) or NAMPT underexpression (sh). The NAMPT-overexpressing cells grew faster than the parental cells, indicating that NAMPT confers a proliferative advantage (Figure 3E–3F), while the NAMPT-underexpressing cells grew slower than the parental cells, confirming the role of NAMPT in tumor proliferation. The NAMPT-overexpressing cells also overcame apoptosis when clonogenic assays were performed at low cell densities (Figure 3G–3H), while the NAMPT-underexpressing cells formed reduced numbers of colonies compared with the parental cells (Figure 3G–3H).

NAMPT overexpression strongly correlated with poor patient prognoses (Figure 2). It has been proposed that CICs are primarily responsible for tumor relapses and poor therapeutic responses, as CICs are able to reconstitute entire tumors.

Therefore, we tested the ability of cells with different levels of NAMPT expression to form tumorspheres, a surrogate assay for the cancer stem-like phenotype [25–28]. The cells were seeded and visualized five days later. The parental cells formed spheres at this stage, which were considered 1st generation tumorspheres. The number of tumorspheres derived from the cells with increased NAMPT expression was significantly higher than that derived from the control cells (Figure 3I), while the cells with decreased NAMPT expression showed a clear reduction in the number of tumorspheres (Figure 3I).

To further explore the cancer-initiating cell properties induced by NAMPT, we cultured cells at low densities to form independent colonies comprising individual clones, which were previously classified as holoclones, meroclones and paraclones based on their ability to reconstitute tumors from a single cell [25–28]. Holoclones are believed to be derived from stem cells, while paraclones are differentiated cells that are incapable of reconstituting a culture [29]. Meroclones are intermediate phenotypes between holo- and paraclones. The percentage of holoclones in NAMPT-expressing cells was increased from 20% to 60% in SF268 (Figure 3J–3K) and from 60% to 80% in U251MG, while the percentage of holoclones was decreased in cells expressing NAMPT shRNA (Figure 3J–3K), indicating a relationship between NAMPT and the CIC phenotype.

In addition, FACS analysis of tumor cells showed higher stem cell phenotypic marker expression, which correlated with NAMPT expression. NAMPT-expressing cells showed increases in both CD133+ (Figure 3L) and CD44+ pools (Figure 3M). However, NAMPT-underexpressing cells showed clear reductions in the CD133+ and CD44+ pools (Figure 3L–3M), confirming that NAMPT is a factor that facilitates tumor cell de- differentiation to a pluripotent-like state. A second shRNA against NAMPT showed similar results (Supplementary Figure 1A–1C).

To finally confirm these data, we tested the ability of cells with different levels of NAMPT expression to form tumorspheres from one isolated single cell, another surrogate assay for the CIC-like phenotype [28, 30–32]. The cells were seeded at one cell per well and visualized 21 days later (Figure 3N). The parental cells formed spheres at this stage, which were considered 1st generation tumorspheres. The number and size of tumorspheres derived from the cells with increased NAMPT expression was significantly higher than that derived from the control and parental cells (Figure 3N–3O). We also explored the effect of a second sh in both cell lines in order to avoid overlapping effects and we found similar results (Supplementary Figure 2).

NAMPT catalyzes the conversion of nicotinamide to nicotinamide mononucleotide (NMN), which is the rate-limiting step in the NAD salvage pathway. If the enzymatic activity of NAMPT is directly responsible for these phenotypes, the phenotype should be recovered by directly adding the product of the enzyme to cells. Therefore, we repeated the previous surrogate experiments and added saturating concentrations of NMN to cells expressing low levels of NAMPT (sh supplemented with NMN, sh+NMN). In these experiments, we rescued the parental phenotypes by supplying the cells with the NAMPT metabolic product NMN (Supplementary Figure 3A–3L left). In all cases, the cells with downregulated NAMPT expression but with abundant NMN in the media behaved similarly to the parental cells. However, they did not exhibit the increased levels of tumorigenicity induced by NAMPT overexpression (Supplementary Figure 3A–3L left). Similar results were found using a second shRNA against NAMPT (Supplementary Figure 4A–4D).

NAMPT is also known to act as an extracellular soluble protein named visfatin, which has activity that is not related to the enzymatic activity of the protein [33, 34]. Therefore, we explored the effects of extracellular visfatin supplementation in NAMPT- underexpressing cells and found that the NAMPT-underexpressing cells were rescued, similar to the above findings (Supplementary Figure 3A–3L right). However, as before, the cells did not exhibit the increased levels of tumorigenicity induced by NAMPT overexpression (Supplementary Figure 3A–3L right). Similar results were found using a second shRNA against NAMPT (Supplementary Figure 4).

These results clearly indicate that NAMPT gene overexpression provides the protein with properties beyond those associated with its enzymatic function in the salvage pathway, conferring CIC-like properties to the cells in which is expressed.

### NAMPT expression correlated with high levels of cancer initiating cell-like cells in glioblastoma directly from patients

**Figure 4:**
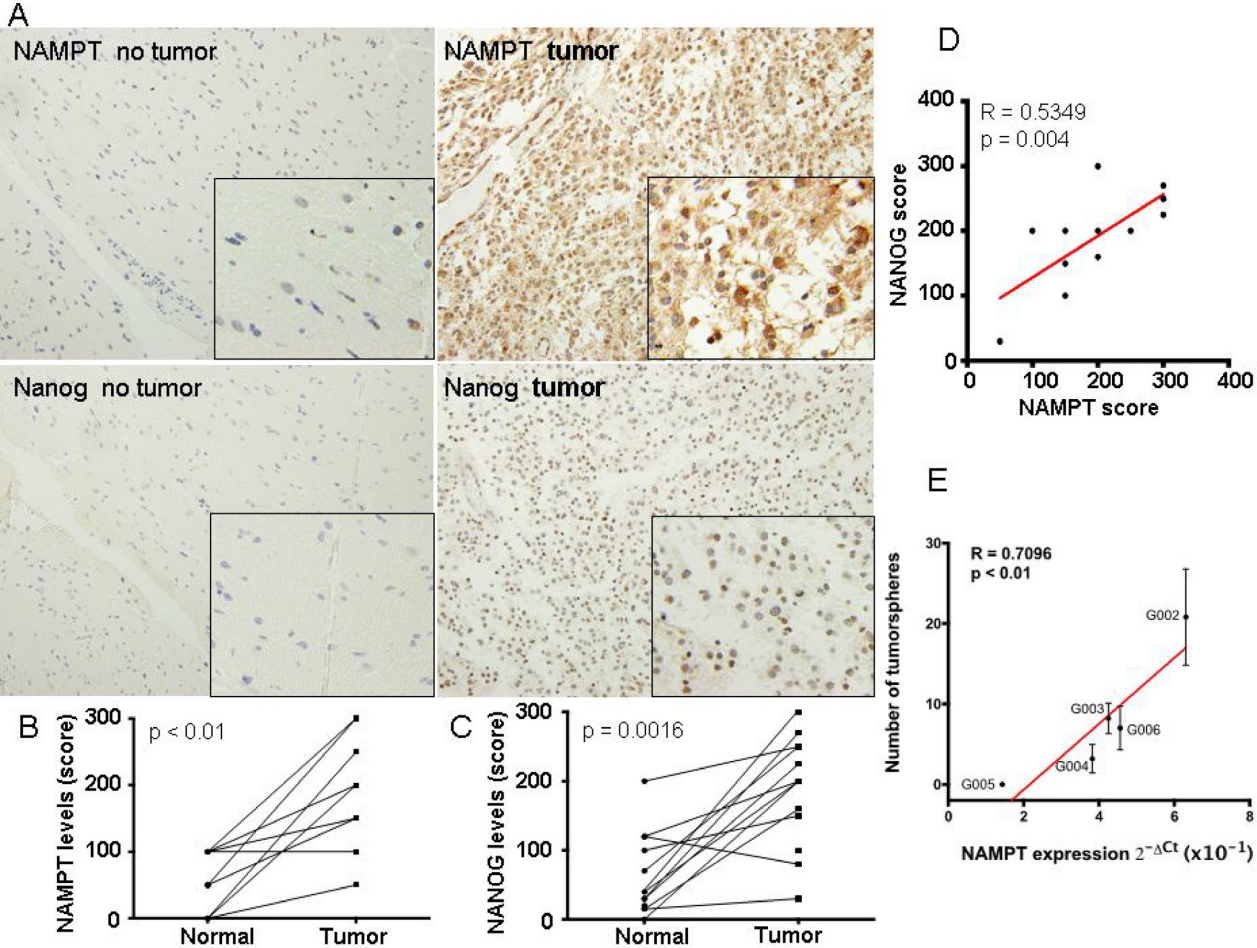
NAMPT expression correlated with high levels of cancer stem cell-like cells in glioblastoma extracted directly from patients (**A**) We first took 14 glioblastoma tumor samples directly from patients as well as matched non-tumor samples and generated a tissue microarray, TMA. These TMAs were stained for NAMPT and Nanog according to M&M. In these samples we evaluated the expression of NAMPT and Nanog, as surrogated marker of cancer stem cell-like levels of these tumors. (**B**) Levels of NAMPT and (**C**) Nanog were related in matched samples, from the same patient. (**D**) Evaluation of the correlation between matched samplesof NAMPT and Nanog. There was a clear correlation (pearson r = 0.53, *p <* 0.01) between the expression of Nanog and NAMPT. (**E**) We took 5 fresh glioblastoma samples from patients. After tissue disaggregation, we directly measured NAMPT levels by Q-RT-PCR and parallely seeded 3000 cells to measure the number of tumorspheres formed. Then we plotted to establish 1 to 1 correlation, between the level of NAMPT and the number of tumorspheres. We found a strong direct correlation of the tumorsphere number and NAMPT levels for each tumor.

To approach our findings to a more *in vivo* situation, we first took 14 glioblastoma tumor samples directly from patients as well as matched non-tumor samples. In these samples we measured the expression of NAMPT and Nanog, as surrogated marker of CIC-like levels of these tumors (Figure 4A). We found a clear increase of NAMPT and Nanog in tumoral vs non-tumoral samples (Figure 4B and 4C). And more importantly, there was a clear correlation (pearson r = 0.53, *p <* 0.01) between the expression of Nanog and NAMPT (Figure 4D). These data reinforce the direct relationship between NAMPT and the cancer stem cell like component of glioblastoma tumors.

Then, we took 5 fresh glioblastoma samples from patients (Supplementary Table 1). After tissue disaggregation, we directly measured NAMPT levels by Q-RT-PCR and in parallel seeded 3000 cells to measure the number of tumorspheres formed (Figure 4E). Then we plotted to establish 1 to 1 correlation, between the level of NAMPT and the number of tumorspheres. We found a strong direct correlation of the tumorsphere number and NAMPT levels for each tumor (Figure 4E, pearson r = 0.709; *p <* 0,01).

All these data strongly confirm that NAMPT levels correlates with the activation of the cancer stem cell–like phenotype in human glioblastoma tumors.

### NAMPT induces pluripotency via signalling pathways controlling stemness

**Figure 5:**
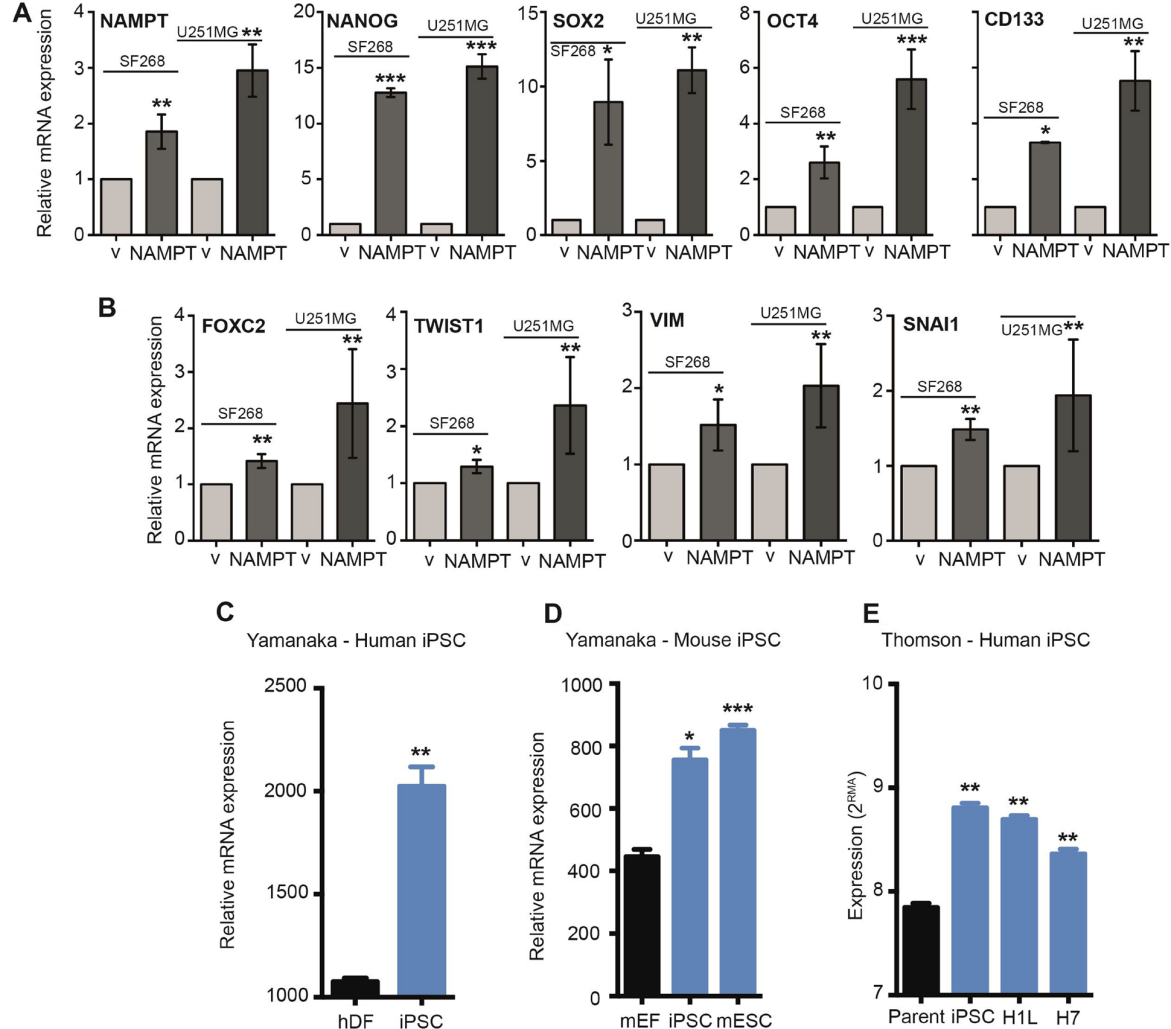
NAMPT activates EMT and stem pathway effectors (**A**) Q-RT-PCR analysis of NAMPT overexpression in empty vector [^**^p < 0.01 with *t*-test]. Q-RT-PCR analysis of stem cell gene expression in empty vector: NANOG [^***^p < 0.001 with *t*-test], SOX2 [^*^p < 0.05; ^**^p < 0.01 with *t*-test], OCT4 [^**^p < 0.01; ^***^, *p* < 0.001 with *t*-test] and CD133 [^*^p < 0.05; ^**^p < 0.01 with *t*-test]. (**B**) RT-qPCR analysis of EMT gene expression in empty vector: FOXC2 [^**^p < 0.01 with *t*-test], TWIST1 [^*^p < 0.05; ^**^p <0.01 with *t*-test], VIM [^*^p <0.05; ^**^p <0.01 with *t*-test] and SNAI1 [^**^p < 0.01 with *t*-test]. (**C**) Analysis of the Yamanaka dataset (GSM241846) with respect to induced pluripotent stem cell (iPSC) generation from human dermal fibroblasts (hDF) demonstrates an increase in NAMPT levels with reprogramming [^*^p = 0.01; ^**^p < 0.01 with ANOVA compared to human dermal fibroblasts]. (**D**) Analysis of the Yamanaka dataset (GSE15148) with respect to iPSC generation from mouse embryonic fibroblasts (mEF) demonstrates a significant increase in NAMPT expression with reprogramming. Mouse embryonic stem cells (mESC) also express high levels of NAMPT compared to fibroblasts [^*^p = 0.01; ^**^p < 0.01 with ANOVA compared to mouse embryonic fibroblasts]. (**E**) Analysis of the Thomson dataset (GSE5259) with respect to iPSC generation from human foreskin fibroblasts (parent) demonstrates an increase in NAMPT levels with reprogramming. Human embryonic stem cell lines (H1L and H7) also express high levels of NAMPT compared to fibroblasts [^*^p = 0.01; ^**^p < 0.01 with ANOVA compared to human foreskin fibroblasts]. V: cells expressing vector only. NAMPT: Cells overexpressing ectopic NAMPT cDNA.

To support this novel finding, we decided to explore the correlation between NAMPT expression and cell stemness. We analyzed whether the stem transcriptional core formed by SOX2, OCT4 and NANOG was altered by NAMPT levels. By Q-RT-PCR, we analyzed each transcript individually, confirmed the overexpression of NAMPT in SF268 and U251MG cells (Figure 5A) and found that NAMPT-overexpressing cells significantly induced Sox2, Oct4 and Nanog mRNA expression (Figure 5A). Furthermore, these cells also showed increased levels of CD133 transcripts (Figure 5A).

The epithelial-mesenchymal transition (EMT) is an essential step mediating tumor reprogramming and metastasis. Several genes are expressed during EMT induction [35, 36]. For the next step in our study, we tested whether NAMPT upregulated some of these steps. We found that NAMPT-overexpressing cells showed induction of FOXC2, TWIST1, VIM and SNAI1 expression (Figure 5B).

Then, we analyzed iPSC transcriptional databases. We found that NAMPT is highly overexpressed in iPSCs and embryonic stem cells (Figure 5C–5E), as they maintain a pluripotent, self-renewed state.

**Figure 6:**
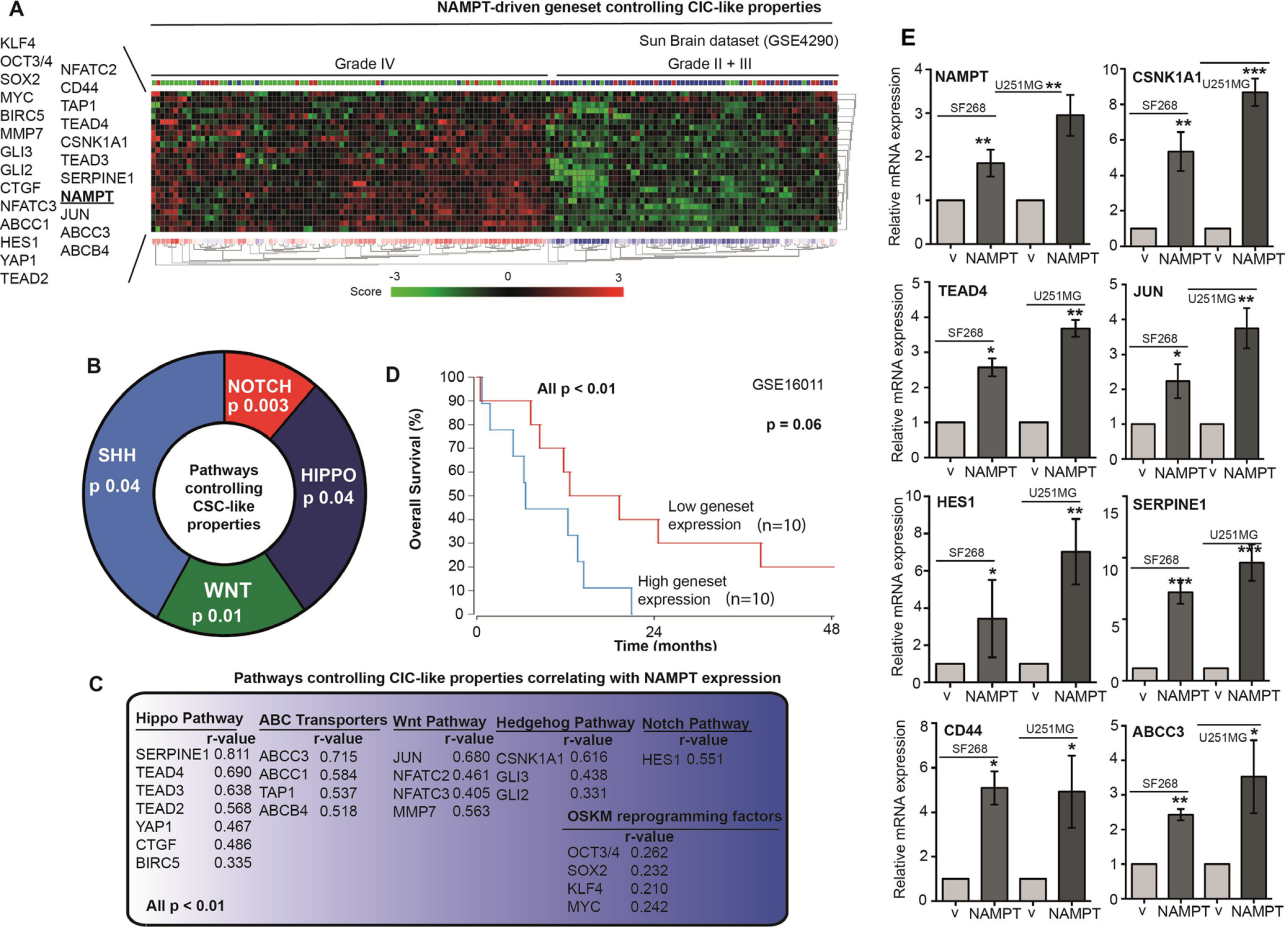
NAMPT induces a gene signature that correlates with glioma tumor grade and predicts poor survival (**A**) R2 expression analysis of the gene signature triggered by NAMPT in the Sun dataset shows a correlation between NAMPT expression and advanced glioma stage: grade IV. R2 expression analysis of the gene signature triggered by NAMPT showed genes related to Stem Cell phenotype. (**B**) pathways controlling CIC-like propersies related to NAMPT expression. (**C**) Genes associated to NAMP gene expression [all *p <* 0.01, Pearson r is shown in each case], and the pathway each gene is associated to. (**D**) Overall survival probability comparing the patients showing low expression signature vs high expression signature. R2 expression analysis of the gene signature triggered by NAMPT in the Sun Brain tumor database in a biased cohort of patients with high signature expression [*n =* 10] and low signature expression [*n =* 10] shows poor survival in patients [*p =* 0.06 with log-rank analysis]. (**E**) Analisis of the expression of the different genes of the signature in SF268 and U2521MG expressing NAMPT.

With the aim of identifying the pathways that induce cell stemness and pluripotency, we correlated NAMPT expression with stem cell pathways in several in silico glioma retrospective studies (GSE16011, GSE4290, GSE4271, GSE43378, and GSE7696). We found a positive correlation with the different stem cell signaling pathways. NAMPT expression showed a strong correlation (pearson r) with Hippo, Wnt, Hedgehog and Notch, as well as ABC transporters as markers of stem functionality (Figure 6A–6C). Furthermore, NAMPT also showed correlation to OSKM reprogramming factors (Figure 6C). The analisis of the geneset comprising all these genes that correlate to NAMPT is highly predictive of patient prognosis (Figure 6D). Therefore, we set to determine whether the different pathways were hyperactivated in NAMPT-overexpressing cells. To this end, we used Q-RT-PCR to measure individually the mRNA levels for Nampt, CD44, Jun, TEAD4, CSNK1A1, ABCC3, Serpine1 and HES1, as the transcriptional markers of the CSC pathways (From Figure 6C). We observed clear transcriptional activation of all these genes in our cells overexpressing NAMPT (Figure 6E). It appears that NAMPT overexpression activates stem cell signaling, eventually activating Hippo, Hedgehog, Wnt and Notch as effectors, contributing to cell stemness.

### NAMPT triggers a gene signature that correlates with poor survival in glioma

**Figure 7:**
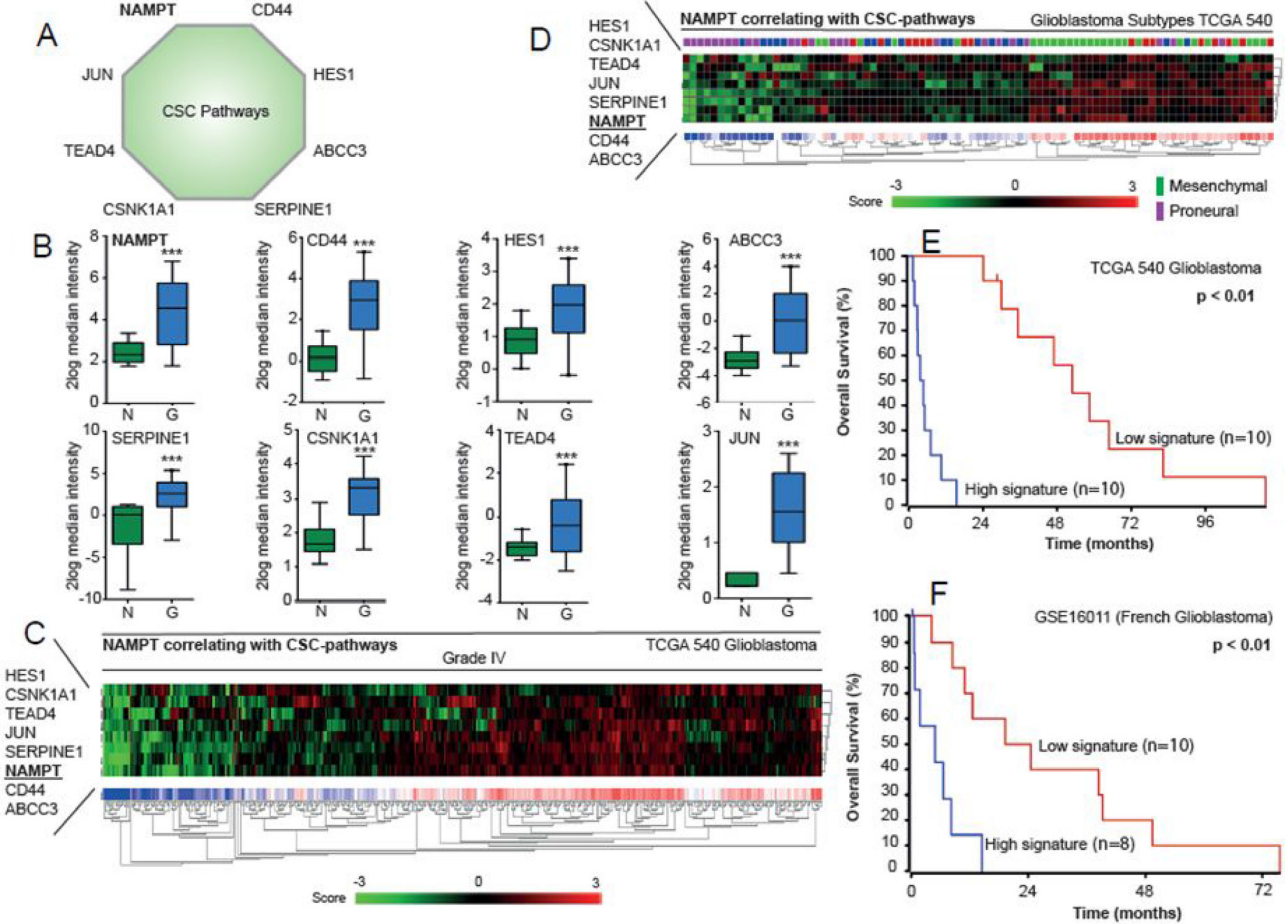
Analysis of NAMPT-related signature in the TCGA database (**A**) Nampt-derived signature is represented. (**B**). Relative expression leves of each gene are represented in normal brain vs glioma samples. (**C**) Nampt-derived signature levels clasiffy Grade IV gliobastoma samples. (**D**) Nampt-derived signature levels classify the different subtypes of Glioblastoma according to the whole dataset of Glioblastoma TCGA dataset. (**E**–**F**) Overall survival probability comparing the patients showing low expression signature vs high expression signature. R2 expression analysis of the gene signature triggered by NAMPT in the TCGA 540 (E) or French (F) glioblastomma databases in a biased cohort of patients with high signature expression [*n =* 10] and low signature expression [*n =* 10] shows poor survival in patients [*p <* 0.01 with log-rank analysis].

NAMPT induces genes associated with the EMT, the Notch pathway and iPSCs, which increase tumorigenicity and expression of the CIC-like phenotype. It is possible that these factors can be used to predict the prognosis of glioma patients. We selected NAMPT and genes activated by its overexpression, including Jun, CD44, HES1, TEAD4, CSNK1A1, ABCC3 and Serpine1 (Figure 7A). All these transcripts were increased in glioblastoma patient tissue samples compared to their corresponding levels in normal brain tissue (Figure 7B). This signature is able to stratify patients with grade IV glioblastoma (Figure 7C). Interestingly, this signature also stratifies patients with different glioblastoma subtypes according to TCGA database, showing higher expression among mesenchimal subtypes, and lower among the proneural subtypes (Figure 7D). The application of the signature clearly distinguishes the patients with good from bad pronosis in the databases applied (Figure 7E–7F).

**Figure 8:**
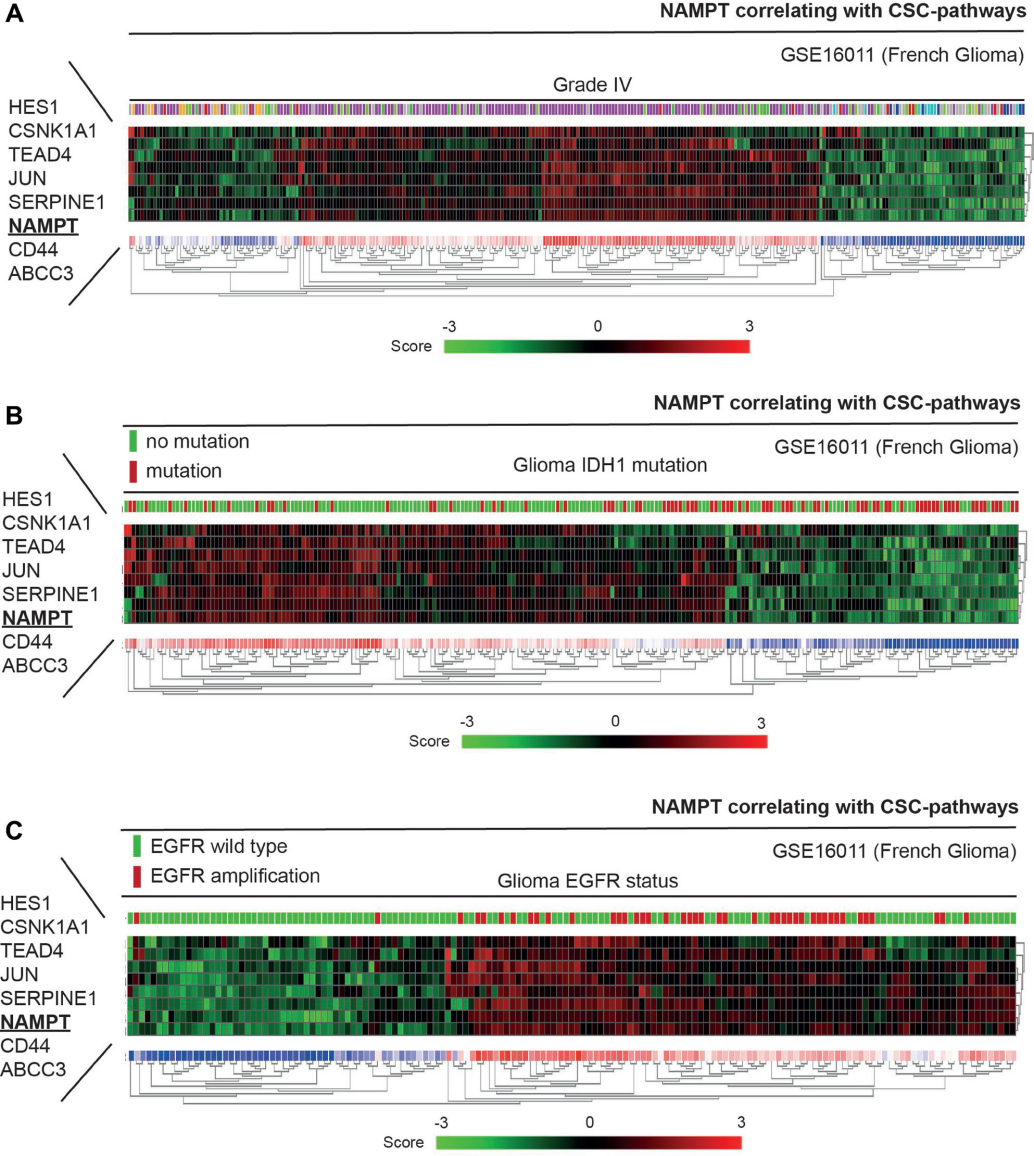
NAMPT induces a gene signature that correlates with WT IDH1 patients and EGFR positive tumors To this study we have selected the French database that contains information about the IDH1 and EGFR mutational status. (**A**) Nampt-derived signature is present in a high percentage of Grade IV gliobastoma samples from French glioma database. (**B**) Nampt-derived signature predicts an enrichment of IDH1 mutatins in patients negative for our signature, indicating a better prognosis in patients with IDH1 mutations. (**C**) On the other hand, most, if not all, gliomas with EGFR amplifications strong positive correlation with our NAMPT-derived signature.

Through the analysis of the NAMPT-derived signature in different datasets, we observed that the incidence of the gene signature was driven by grade in brain tumor patients (Figures 7C–7D and 8A). The percentage of patients who showed a clear positive NAMPT-derived signature increased along with tumor grade in the different datasets analyzed (Supplementary Figures 5–7), exceeding 50% among patients with grade IV tumors. Furthermore, comparisons of Heat map plots between the groups with grade III and IV tumors with high expression of the signature and the groups with grade IIII and IV tumors with low expression of the signature showed that patients with a positive signature have a poor prognosis (Supplementary Figures 5–7). Therefore, the NAMPT-derived signature is capable of accurately predicting poor patient outcomes independently of tumor grade.

Finally, we analyzed the correlation of our NAMPT-derived signature with other proposed mutations that have been proposed to be drivers of the malignant status of glioblastoma tumors [37, 38]. We found that our signature predicts an enrichment of IDH1 mutations in patients negative for our signature, indicating a better prognosis in patients with IDH1 mutations (Figure 8B, Supplementary Figure 8). On the other hand, most, if not all, gliomas with EGFR amplifications showed strong positive correlation with our NAMPT-derived signature (Figure 8C, Supplementary Figure 8).

Therefore, we confirmed that NAMPT is a potent oncogene that confers CIC-like properties, which are responsible for the poorer responses and patient prognoses associated with glioblastoma.

### NAMPT is a suitable target on glioma CICs

NAMPT is essential for biosynthesis of NAD. Inhibition of NAMPT may lead to depletion of NAD+, which in turn inhibits ATP synthesis. This effect eventually causes attenuation of cancer cell proliferation and death. Therefore, NAMPT was proposed as an interesting therapeutic target. Our data support this idea, particularly with respect to brain tumor patients with a NAMPT signature, as these patients have poor prognoses.

FK866, a NAMPT inhibitor [20, 21], was tested as a possible therapy for gliomas in mass cultures and tumorspheres representing CIC populations. NAMPT-overexpressing cells were more sensitive to FK866 in mass cultures than parental cells and NAMPT- underexpressing cells. As standard treatment, Temozolomide (TMZ) toxicity was also assessed both individually and in combination with FK866 (Table 1). We assessed FK866-induced toxicity in tumorspheres and observed that FK866 was effective against this CIC population (Table 1). Interestingly, the combined treatment with TMZ slightly sensitized these values, both in mass cultures and tumorspheres suggesting this combination as an effective therapy (Table 1, Supplementary Figure 9). Thus, we believe that NAMPT inhibitors, in combination with TMZ may represent a new therapy for glioma CIC populations, particularly in patients expressing high levels of the gene signature.

**Table 1:** NAMPT is a suitable target in both monolayer tumor cells and tumorspheres cytotoxic analysis of the NAMPT inhibitor FK866 and TMZ shows IC50s of SF268 and U251MG alone or in combination in monolayer culture or Tumorsphere specific assays

	Monolayer - Monotherapy			
	SF268		U251MG	
	FK866 (nM ± SD)	TMZ (μM ± SD)	FK866 (nM ± SD)	TMZ (μM ± SD)
*sh*	8.3 ± 0.8	163.2 ± 2.2	22.3 ± 0.6	203.5 ± 4.8
*v*	5.2 ± 0.9	165.4 ± 1.9	20.7 ± 0.7	194.1 ± 3.4
*NAMPT*	2.8 ± 0.3	173.6 ± 2.1	15.3 ± 1.2	201.4 ± 5.3

	Tumorspehres - Monotherapy			
	SF268		U251MG	
	FK866 (nM ± SD)	TMZ (μM ± SD)	FK866 (nM ± SD)	TMZ (μM ± SD)
*sh*	9.5 ± 0.9	174.1 ± 3.6	27.3 ± 1.4	221.4 ± 3.2
*v*	6.1 ± 1.2	169.8 ± 2.1	18.6 ± 1.9	224.5 ± 1.7
*NAMPT*	2.5 ± 0.4	173.3 ± 3.2	11.3 ± 1.3	221.6 ± 4.7

	Monolayer - Combined	
	SF268	U251MG
	FK866 + 100 μM TMZ (nM ± SD)	FK866 + 150 μM TMZ (nM ± SD)
*sh*	6.1 ± 0.2	19.4 ± 0.7
*v*	2.4 ± 0.4	14.3 ± 1.3
*NAMPT*	0.2 ± 0.4	5.1 ± 1.4

	Tumorspheres - Combined	
	SF268	U251MG
	FK866 + 100 μM TMZ (nM ± SD)	FK866 + 150 μM TMZ (nM ± SD)
*sh*	7.3 ± 0.7	25.4 ± 1.8
*v*	4.2 ± 0.3	17.1 ± 0.9
*NAMPT*	0.5 ± 1.1	7.2 ± 0.7

For the assay, 5 × 10^3^ cells were seeded and then treated with the different compound at 11 different concentrations at 1/3 dilutions after 24 hours. Then, 96 hours later, cell viability was measured via MTT assay and validated independently by crystal violet staining. IC50 was calculated as the concentration allowing 50% survival compared to day 0 controls. Combination experiments were performed testing 11 different concentrations at 1/3 dilutions of FK866 and maintaining same suboptimal concentration of TMZ (as indicated) in all tested points.

## DISCUSSION

Gliomas are malignant tumors associated with poor prognosis and low median survival time. Among them, glioblastoma is the most common malignant primary brain tumor in adults and one of the most lethal human cancers [38–40]. Glioblastoma is not a surgically curable disease because the tumor cells invade the surrounding brain tissue and are among the most resistant to radiation and cytotoxic chemotherapy [41, 42]. Therefore, new advances in stratification strategies, molecular knowledge and treatment approaches are needed. In 2010, Verhaak RG *et al* set the basis of a molecular categorization of Glioblastomas [37], later used by the TCGA. Later studies based on tumor sequencing analyses from different tumor areas and paired biopsy at diagnosis and recurrent tumor analyses have shown that multiple subtypes can co-exist within the same tumor and can change after treatment. We found that NAMPT is particularly overexpressed in 3 out of these 4 subtypes. We found that our signature predicts an enrichment of IDH1 mutations in patients negative for our signature, indicating a better prognosis in patients with IDH1 mutations [43]. On the other hand, most, if not all, gliomas with EGFR amplifications showed strong positive correlation with our NAMPT-derived signature.

Here, we showed that the NAMPT gene is highly overexpressed in a large percentage of glioma tumors, in accordance to Gujar *et al* recently [44]. This percentage increases in late-stage tumors. Furthermore, tumors with high NAMPT expression levels were associated with poor prognosis, independently of tumor stage. Ectopic overexpression of NAMPT in glioma cells increases its protumorigenic properties, as well as its cancer initiating cell-like physiological properties. However, downregulation of NAMPT via overexpression of specific shRNAs reduces its tumorigenicity and CIC-like properties. We showed that NAMPT activates Stemness maintenance and EMT pathways, as demonstrated by the activation of several transcriptional markers. NAMPT also activates OSKM factors by activating SOX2, OCT4 and Nanog. Therefore, NAMPT overexpression facilitates self-renewal cell properties, resulting in stemness-like maintenance, which ultimately leads to increased migration, de-differentiation and CIC-dependent resistance to therapy, which are features of glioma tumors.

Nicotinamide phosphoribosyl transferase, NAMPT, catalyzes the conversion of nicotinamide to nicotinamide mononucleotide (NMN), which is the rate-limiting step in the NAD salvage pathway. NAMPT is essential for biosynthesis of NAD and has been found to be upregulated in many cancer cells [13, 14, 33, 44]. Inhibition of NAMPT can lead to depletion of NAD+, which in turn inhibits ATP synthesis. This effect eventually causes attenuation of cancer cell proliferation and death [18–21, 45]. Metabolic switch is a key factor in cellular transformation. Human cancers exhibit altered metabolism and depend heavily on glycolysis, the Warburg effect [46]. Due to the abnormal consumption of glycolytic end-products and NAD+, tumors have dramatically increased glucose needs [13, 14, 44]. This abnormal metabolism is enhanced in CICs. Our study showed that NAMPT facilitates increases in CIC subsets, defining a novel signature characterized by early development and stem-like property maintenance. Our results suggest that CICs in primary gliomas primarily use the NAD salvage pathway to maintain a full, rich source of nutrients for enzymatic activity, leading to tumor progression and eventual reprogramming, which ultimately results in relapse. Our data also suggest that CICs have a competitive advantage in any nutrient-limiting microenvironment. NMN and visfatin, when supplied to NAMPT-underexpressing cells, restored the phenotype of the parental cells. NMN may difuse through the membrane supplying the NMN deficient production by NAMPT downregulation. Visfatin, as extracellular NAMPT may act biochemically metabolizing extracellular nicotinamide into NMN which may diffuse to the cell, restoring the parental phenotype.

NAMPT-driven stemness is closely associated with upregulation of the Stem cell pathways signaling pathways. Recent studies [43, 44] have highlighted the importance of these pathways, which mediated tumor chemoresistance, and its upregulation has been linked to radiotherapy resistance in tumor cells derived from glioma CICs. Our data are in accordance with those of most previous works indicating that stem cell pathways upregulation has greater tumorigenic potential in human cancer and that a positive correlation exists between glioma stage and NAMPT expression.

Based on the above findings, we explored whether a NAMPT-dependent profile comprising Nampt, CD44, Jun, TEAD4, CSNK1A1, ABCC3, Serpine1 and HES1 enables correct stratification of glioblastoma patients. Our data clearly showed that this NAMPT-dependent profile correctly separated patients with good prognosis from those with bad prognosis. Altogether, our data suggest that NAMPT may be a suitable therapeutic target for glioblastoma, especially in patients with poor prognosis. Because NAMPT induces the CIC phenotype, we tested whether tumorspheres, as *in vitro* surrogates for tumor CICs, respond to the NAMPT inhibitor FK866. We found that this toxicity driven by NAMPT inhibitor could be strengthened in combination with TMZ. We found that glioma tumorspheres are sensitive to NAMPT inhibitors, particularly tumorspheres with high levels of NAMPT expression, which indicates that NAMPT inhibition may be a suitable therapy for glioblastoma. It has been recently demonstrated that IDH1 mutant gliomas respond to NAMPT inhibition [47]. We found that IDH wild type correlated to NAMPT overexpression (Figure 8B). Since, cells overexpressing NAMPT gene, are more sensitive to its inhibition, either by NAMPT inhibition alone or in combination with temozolamide (Table 1), this data suggest that NAMPT inhibition effect is independent of the IDH1 mutations and dependent only on the levels of NAMPT expression. Although NAMPT is expressed in a wide range of normal tissues, the brain is a very metabolically active organ, which may represent a therapeutic window for drug targeting. Furthermore, unlike glioma cells, healthy neurons remain in a quiescent, post- mitotic state, suggesting that anti-NAMPT combination therapies with conventional drugs that target rapidly proliferating cells may have effects on CICs while sparing neurons, supporting the use of NAMPT inhibitors in cancer treatment.

## MATERIALS AND METHODS

### Cell culture and cell transfection

The human Glioblastoma cell line SF268 and U251MG were cultured in RPMI 1640 medium (SIGMA) supplemented with 10% Fetal Bovine Serum (FBS) at 37°C under 5% CO_2_ atmosphere. For DNA transfection, SF268 and U251MG were transfected with Mirus TransIT-X2 Dynamic Delivery System (Mirus MIR6000) in exponential phase with 2.5 µg of a small hairping RNA (shRNA) against NAMPT (QIAGEN SureSilencing shRNA insert sequence: 5′-AAGATCCTGTTCCAGGCTATT-3′), 2.5 µg of DNA carrying an hygromycin empty vector (QIAGEN non-coding sequence: 5′-GGAATCTCATTCGATGCATAC-3′) and 2.5 µg of DNA of pCMV-hygromycin carrying a cDNA of NAMPT gene (SinoBiological HG10990-M; NCBI RefSeq: NM_005746.2).

Proliferation assay. A time course curve of parental (empty vector transfected cells) and NAMPT both cDNA and shRNA-expressing cells was generated by seeding 10^3^ cells in 2.2 cm bottom-well diameter dishes in triplicate samples. After 24 h, medium was changed (day 0) and the indicated culture media added. Every 24 hours, cells were fixed and stained with crystal violet 1% (SIGMA C6158-50G). After extensive washing, crystal violet was solubilized in 20% acetic acid (Sigma) and quantified at 595 nm absorbance as a relative measure of cell number (BIORAD iMark™ Microplate Reader). Values are expressed as the percentage of cell growth of cells growing in the presence of 10% FBS. Zero percent refers to the number of cells at day 0.

Cytotoxicity assay. For the assay, 5x103 cells were seeded and then treated with the different compound at 11 different concentrations at 1/3 dilutions after 24 hours. Then, 96 hours later, cell viability was measured via MTT assay and validated independently by crystal violet staining as previously described [48]. IC50 was calculated as the concentration allowing 50% survival compared to day 0 controls.

### Colony formation assay and clonal heterogeinity analysis

10^3^ cells were seeded in 10 cm plates, every condition in triplicate. The medium was replaced every 3 days and after 12 days the colonies were fixed and stained with a Crystal violet assay. After extensive-washing, colonies were counted. Values are expressed in number of observed colonies among 10^3^ seeded cells. To analyze the clonal heterogeneity, 10^2^ random colonies were classified in triplicate depending on its phenotype: Holoclone, Meroclone and Paraclone [29].

### Sphere-forming assay

A total of 5x10^3^ cells/mL/well were seeded in Ultra-low Attachment Plates containing MammoCult™ Basal Medium (Human) (Stem Cell Tech) supplied with 0.48 mg/mL hydrocortisone, 0.2% heparin solution and 10% MammoCultTM Proliferation Supplement (Human). After 4 days, primary tumorspheres were measured, both in size and in number, using an inverted microscope (Olympus CKX41).

### Single cell sphere-forming assay

Single cells were individually seeded through cell sorting (BD FACS Jazz) in 96 well Ultra-low attachment plates containing MammoCult™ Basal medium (Human) (Stem Cell Tech) supplied with hydrocortisone 0.48 mg/mL, Heparin solution 0.2% and 10% MammoCult™ Proliferation Supplement (Human). After 21 days, primary tumorspheres formed were measured both in size and number using inverted microscope (Olympus CKX41).

### Fluorescence-activated cell sorting (FAC)

For flow cytometry analysis, the cell lines were harvested wit 0.25% trypsin, 0.02% EDTA (Sigma Aldrich). The cells were resuspended in cell culture media and were then counted and washed in PBS with 2% FCS and 5 mM EDTA and collected by centrifugation. The cells were blocked with FcR-Blocking reagent human (Miltenyi Biotec) for 10 min on ice. Afterwards, the cells were stained with CD133/1-PE (Miltenyi Biotec) or CD44 (AC-CD44-PE, Miltenyi) at 4°C for 30 min in the dark. Following antibody labeling, the cells were washed twice with PBS with 2% FCS and 5 mM EDTA. Finally, the cells were resuspended in PBS with 2% FCS and 5 mM EDTA. Data were acquired on a FACSCanto II (BD Biosciences, San Jose, USA) and analyzed with Diva-BD software.

### Quantitative RT-PCR

Total cellular RNA was isolated with the RNeasy kit (Qiagen) and reversed transcribed into cDNA using 2.0 µg of RNA, random primers, dNTP mix and a Multiscribe Reverse Transcriptase in a total volume of 50 µL (High Capacity Transcription Kit-Applied Biosystems). Real time PCR was performed on an Applied Biosystem 7900HT cycler using gene-specific probes from Life technologies as follows: NAMPT (Hs00237184_m1), SNAI1 (Hs00195591_m1), TWIST1 (Hs01675818_s1), FOXC2 (Hs00270951_s1), VIM (Hs00185584m1), NANOG (Hs04260366_g1), BMI-1 (Hs00995536_m1), SOX2 (Hs01053049_s1), OCT4 (Hs00999632_g1), SERPINE1 (Hs00167155_m1), CD133 (Hs01009257_m1), ABCC3 (Hs00978452_m1), CSNK1A1 (Hs00793391_m1), HES1 (Hs00172878_m1), TEAD4 (Hs01125032_m1 ), JUN (Hs01103582_s1), CD44 (Hs01075864_m1) and GADPH (Hs03929097_g1) as housekeeping. Relative quantitation values were expressed as 2^-ÄCt^ or relative mRNA expression.

### Immunoblotting

Cells were were lysed in RIPA buffer (Tris–HCl pH 8.0 25 mM, NaCl 150 mM, NP40 1%, Sodium desoxycholate 1%, SDS 1%, Na_3_VO_4_ 1 mM, EDTA 0.5 M, complete protease and phosphatase inhibitor cocktail, 2 mM) and then subjected to 3 sonication cycles during 5 seconds. The amount of protein was determined by Bradford assay using BSA (bovine serum albumin) as a standard. The primary antibodies were purchased from commercial sources as follows: á-tubulin 1:10000 (SIGMA – T9026), NANOG (ab80892 Rabbit polyclonal, Abcam); NAMPT (Anti-Visfatin antibody BETHYL A300-779A),. The secondary antibodies used were: Goat pAB to Rabbit IgG (HRP) 1:5000 (ABCAM ab97051), Rabbit pAB to Mouse IgG (HRP) 1:5000 (ABCAM ab97046).

Cytotoxic Assay MTT. 5x10^3^ SF268 cells were seeded then treated with FK866 24 hours later. After 96 hours, cell viability was measured with MTT.

### Immunohistochemistry

Paraffin blocks were cutted into 2.5ìm sections, mounted and dried on glass slides. Sectioned tissues were deparaffinized in xylol, followed by dehydration in graded alcohol solutions and were stained with hematoxylin-eosin (H&E). Epitope antigen retrieval was performed in sodium citrate (pH 6.5). Endogenous peroxidase activity was blocked using DAKO blocking solution for 20 minutes at room temperature. Non specific protein binding was saturated using PBS+10% FBS, 1% BSA and 0.3% Triton X-100 for 1h at room temperature. The primary antibodies (NANOG abcam ab80892; NAMPT Bethyl A300-372A) were incubated overnight at 4°C. A secondary antibody anti-goat (ab97100) was applied for 1h at room temperature, and the immunocomplexes were revealed using substrate buffer and chromogen (Envision, Flex DAKO). The tissues were counterstained with hematoxylin (DAKO), rehydrated in a graded alcohol series, and mounted using coverslips.

### Retrospective analysis of NAMPT gene expression in human gliomas

Correlations between glioma grade, patient survival, tumor recurrence and NAMPT gene expression were determined through analysis of French, TGCA, French-Core Exon, Sun Brain and Freije datasets respectively which are available through Oncomine (Compendia Biosciences, www.oncomine.org) and R2: Genomics analysis and visualization platform (http://r2.amc.nl/ ). High and low groups were defined as above and below the mean respectively. For analysis with high and low groups, high was defined as greater than one standard deviation above the mean, low is greater than one standard deviation below the mean. The National Cancer Institute’s Repository for Molecular Brain Neoplasia Data (REMBRANDT, http://rembrandt.nci.nih.gov ) was also evaluated for correlations between glioma patient survival and gene expression with up- or downregulation being defined as a 2 fold change relative to mean values.

### Statistical analysis

All grouped data are presented as mean ± standard error. Difference between groups was assessed by ANOVA or Student’s *t*-test using GraphPad Prism software. For survival analysis, Kapalan Meier curves were generated using Prism software and R2 Kaplan Meier plotting service and log Rank analysis performed. All experiments were repeated in each condition in at least duplicate with triplicate technical replicates. Data distribution was assumed to be normal but this was not formally tested. Data obtained for retrospective analysis were collected and processed in appropriate experimental arms.

## SUPPLEMENTARY MATERIALS FIGURES AND TABLE


