# Supplementary material for: NAMPT overexpression induces cancer stemness and defines a novel tumor signature for glioma prognosis
Source: Oncotarget. 2017 Aug 28;8(59):99514–30. doi: 10.18632/oncotarget.20577 (PMC5725111; doi:10.18632/oncotarget.20577)
Supplement: Supplementary file 1 [file oncotarget-08-99514-s001.pdf]

NAMPT overexpression induces cancer stemness and defines a novel tumor signature for glioma prognosis

SUPPLEMENTARY MATERIALS

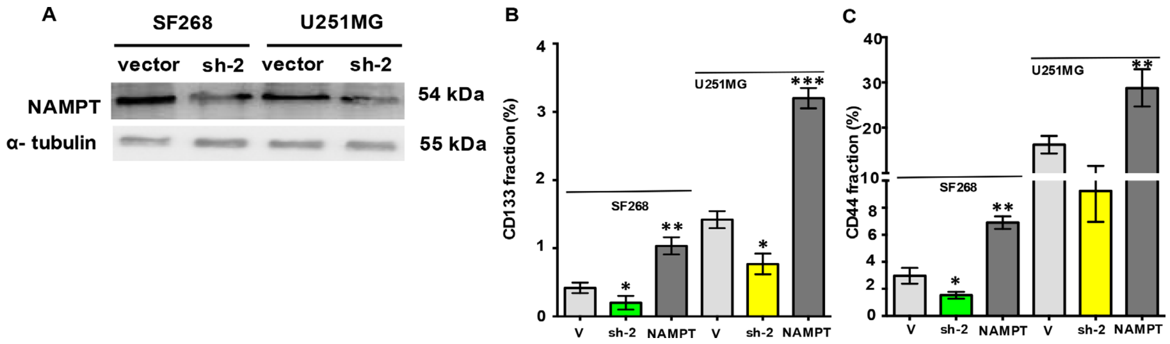

Supplementary Figure 1: CICs related features associated to the causal effect of a second sh expressed on SF268 and U251MG cell lines. We show similar results to the sh-1 using a sh-2 (**A**) in CICs related properties such CD44 and CD133 levels. (**B**) Effect of the second independent Nampt-shRNA on the CD133 positive pool of cells in the population of the cell lines analyzed. CD133 analysis with FACS indicates that NAMPT overexpression increases CD133 levels [$^{*}p < 0.05$ with ANOVA compared to vector], whereas NAMPT underexpression decreases CD133 levels [$^{**}p < 0.01$ with ANOVA compared to vector]. Complementary to Figure 3L. (**C**) Effect of the second independent Nampt-shRNA on the CD44 positive pool of cells in the population of the cell lines analyzed. CD133 analysis with FACS indicates that NAMPT overexpression increases CD44 levels [$^{*}p < 0.05$ with ANOVA compared to vector], whereas NAMPT underexpression decreases CD44 levels [$^{**}p < 0.01$ with ANOVA compared to vector]. Complementary to figure 3M.

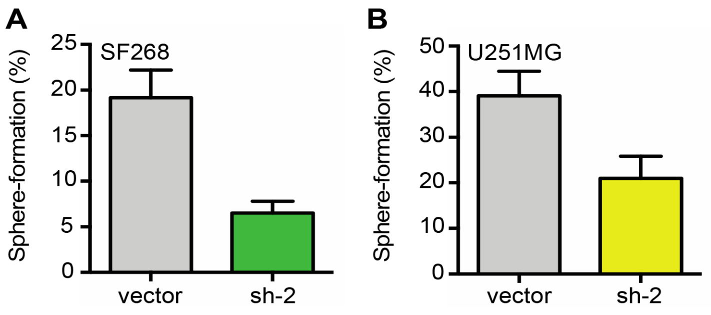

Supplementary Figure 2: Sphere-forming efficiency associated to the causal effect of a second sh expressed on SF268 and U251MG cell lines. We show similar results to the sh-1 using a second sh-2 against NAMPT. We measured the sphere-forming capacity from a single cell on SF268 (**A**) and U251MG (**B**) showing similar results to the sh-1

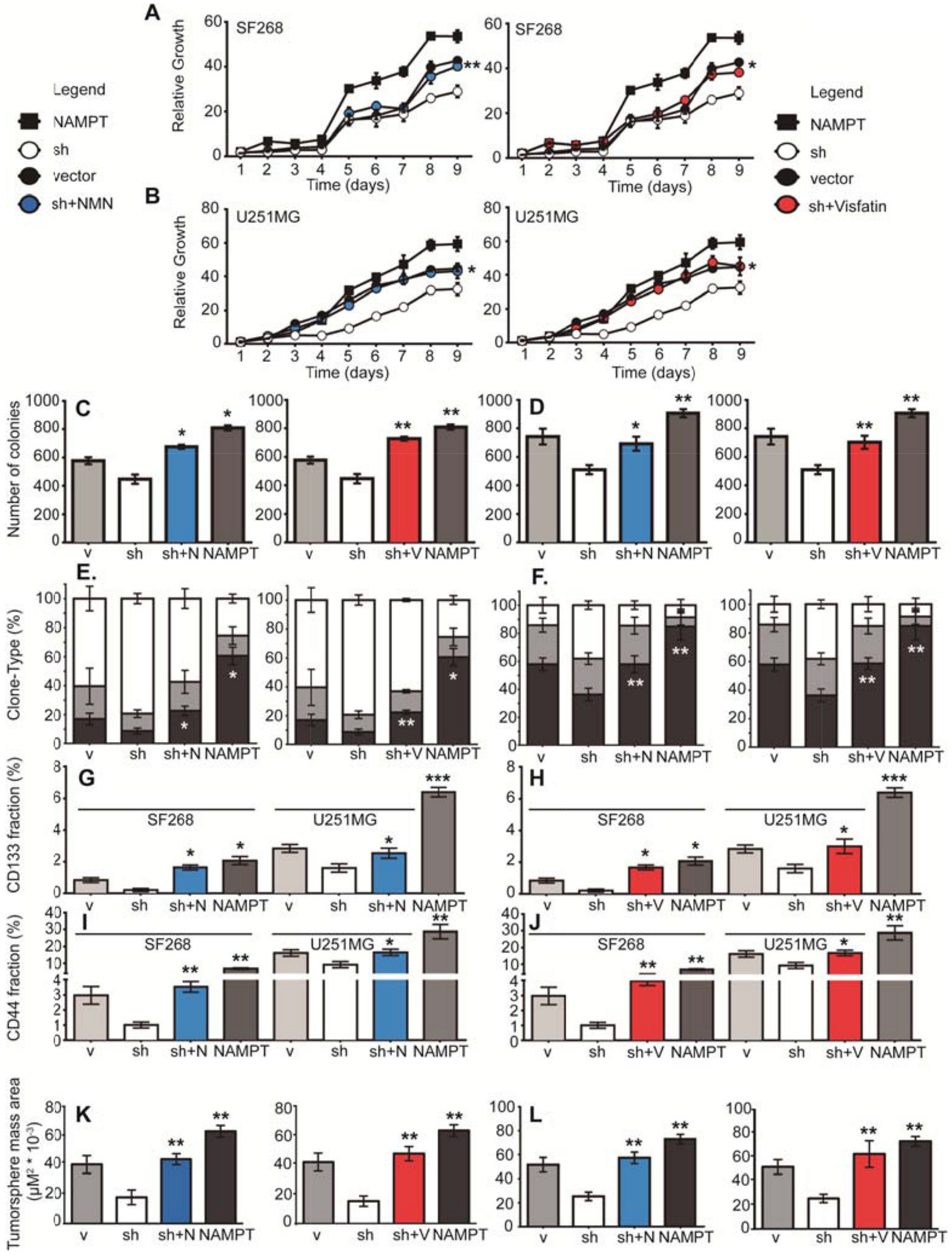

Supplementary Figure 3: NAMPT-driven tumorigenic and CIC properties are rescued both metabolically and enzymatically. (**A**) left - Analysis of the growth curve for SF268 NAMPT-underexpressing cells indicates that 1 mM NMN can rescue the vector-proliferation ratio [**p < 0.01 with ANOVA compared to sh]. right - 60 ng/mL visfatin has the same effect [$^{*}p < 0.05$ with ANOVA compared to sh]. (**B**) Same results described for U251MG [*p < 0.05 with ANOVA compared to sh] (**C**) left - Analysis of clonogenicity in SF268 NAMPT-underexpressing cells indicates that 1 mM NMN can rescue the clonogenetic ability of the vector [$^{*}p < 0.05$ with ANOVA compared to sh] right - 60 ng/mL visfatin has the same effect [$^{**}p < 0.01$ ANOVA compared to sh]. (**D**) Same results described for U251MG [$^{*}p < 0.05$; $^{**}p < 0.01$ with ANOVA compared to sh] (**E**) Analysis of clone phenotypes in NAMPT- underexpressing cells indicates that 1 mM NMN [$^{*}p < 0.05$; $^{**}p < 0.01$ with ANOVA compared to sh] and (**F**) 60 ng/mL visfatin [$^{**}p < 0.01$ with ANOVA compared to sh] can rescue the number of holoclones in the vector in both cell lines. (**G**) CD133 analysis of SF268 and U251MG NAMPT-underexpressing cells indicates that 1 mM NMN [$^{*}p < 0.05$; $^{***}p < 0.001$ with ANOVA compared to sh] and (**H**) 60 ng/mL visfatin [$^{*}p < 0.05$; $^{***}p < 0.001$ with ANOVA comparison to sh] can rescue and surpass the levels of CD133 expression in the vector of both cell lines. (**I**) CD44 analysis of low SF268 and U251MG NAMPT cells indicates that 1 mM NMN [$^{*}p < 0.05$; $^{**}p < 0.01$; $^{***}p < 0.001$ with ANOVA compared to sh] and (**J**) 60 ng/mL visfatin [$^{*}p < 0.05$; $^{**}p < 0.01$with ANOVA compared to sh] can rescue the levels of CD44 expression in the vector of both cell lines. (**K**) left - Analysis of tumorsphere area in SF268 NAMPT-underexpressing cells indicates that 1 mM NMN [$^{**}p < 0.01$ with ANOVA compared to sh] and (K) - right 60 ng/mL visfatin [$^{**}p < 0.01$ with ANOVA compared to sh] can rescue the number of tumorspheres generated in the vector. (**L**) Similar results described for U251MG. V: cells expressing vector only. NAMPT: Cells overexpressing ectopic NAMPT cDNA, Sh: cells expressing an specific NAM shRNA.

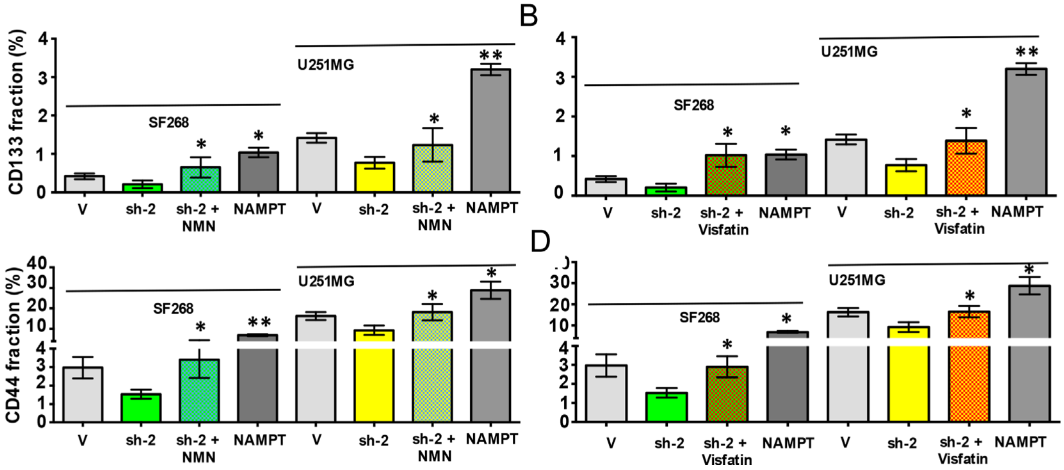

Supplementary Figure 4: NAMPT-driven tumorigenic and CIC properties are rescued both metabolically and enzymatically, using a second shRNA.

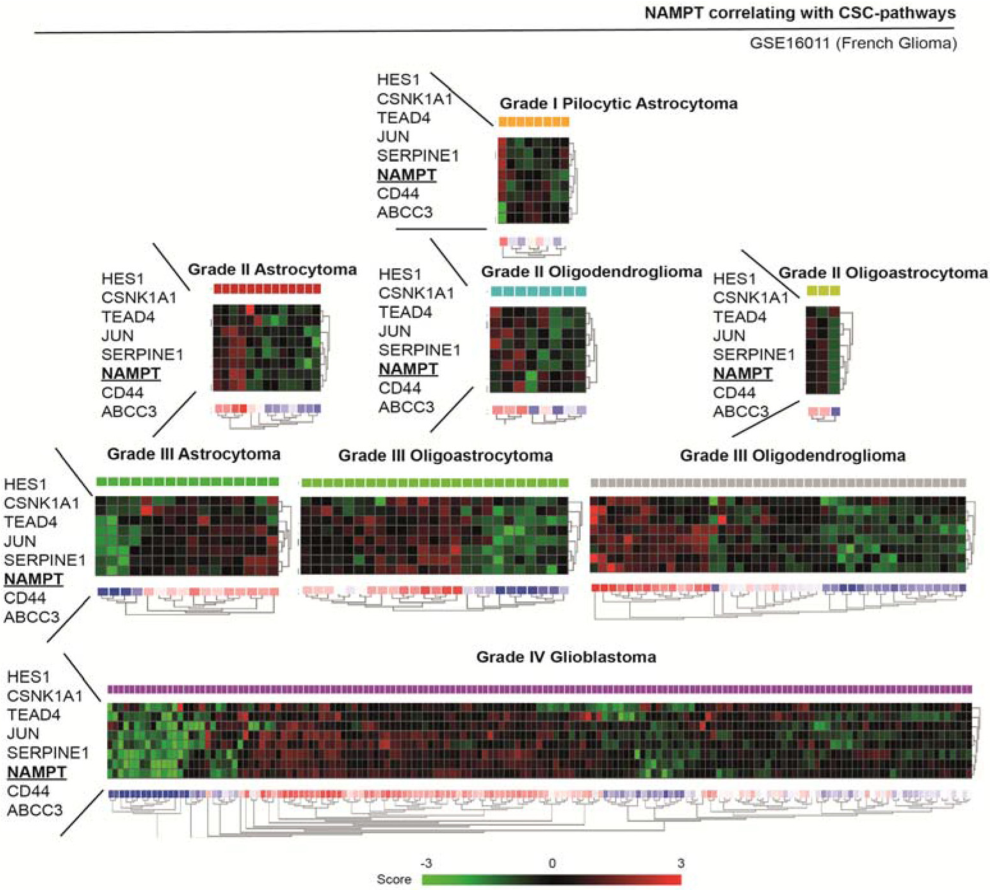

Supplementary Figure 5: Analysis of GSE16011 (French Database) of glioma tumors. Figure shows heat map of selected patients according to tumor grade. In the heat maps, tumors are distributed according to the Nampt-derived signature.

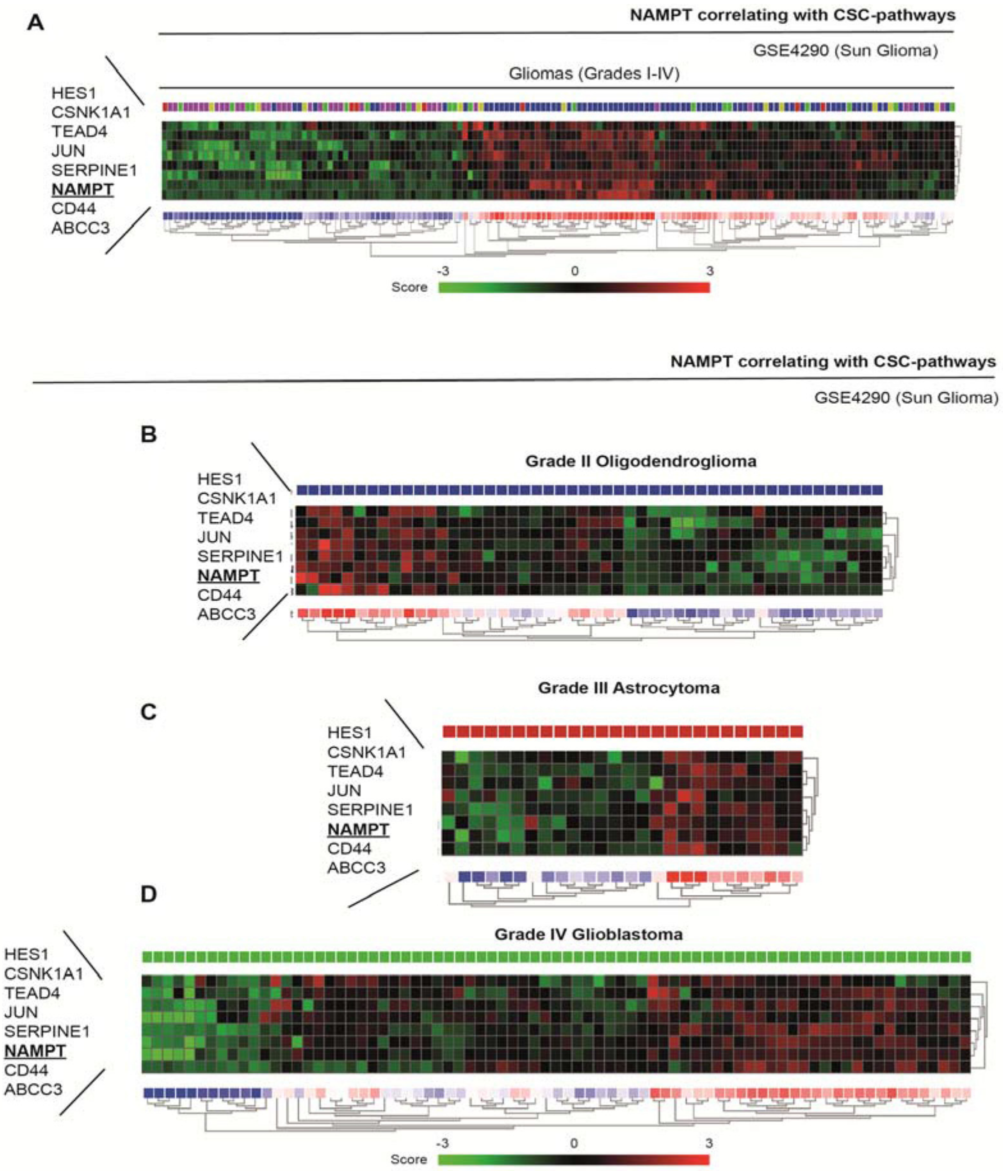

Supplementary Figure 6: Analysis of GSE4290 (Sun Database) of glioma tumors. Figure shows heat map of selected patients according to tumor grade. In the heat maps, tumors are distributed according to the Nampt-derived signature.

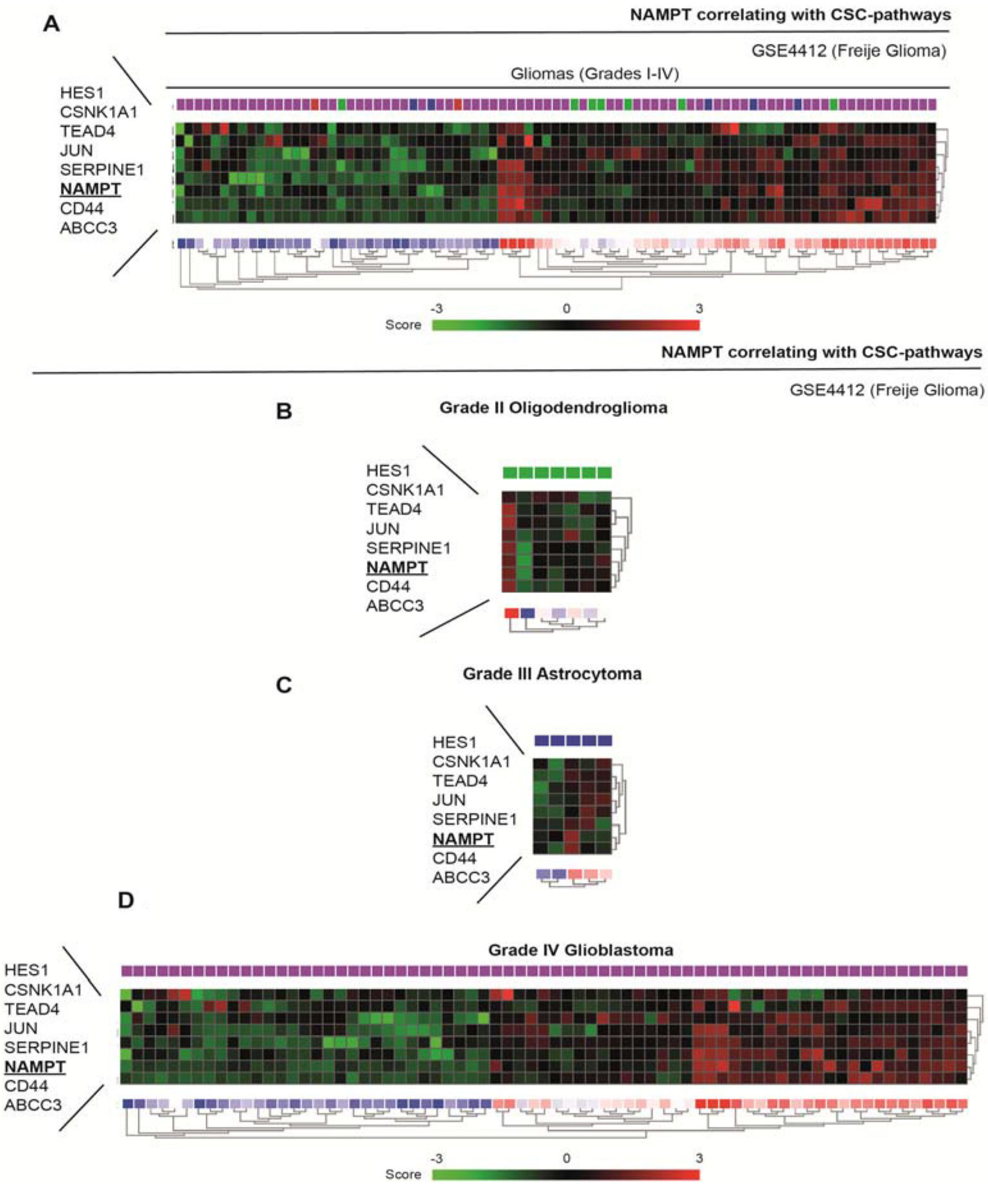

Supplementary Figure 7: Analysis of GSE4412 (Freije Database) of glioma tumors. Figure shows heat map of selected patients according to tumor grade. In the heat maps, tumors are distributed according to the Nampt-derived signature.

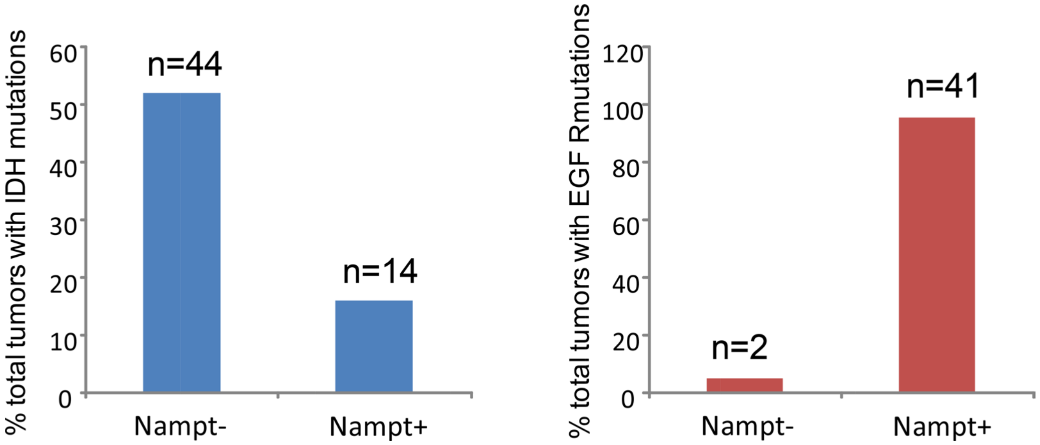

Supplementary Figure 8: Percentage of total tumors with mutations in IDH1 or EGFR mutations or amplifications according to NAMPT levels (Based on figure 8 database).

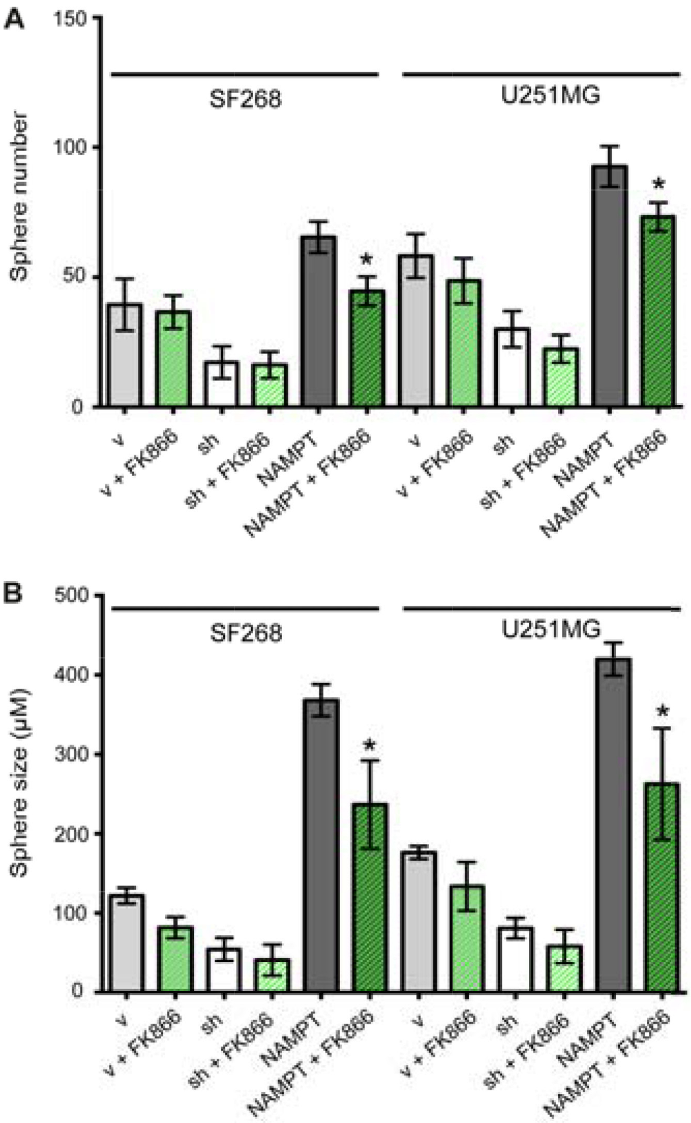

Supplementary Figure 9: The graph shows the average of number of spheres after 4 days of the treatment (+ Std Dev). Treatment indicated was at single dose only (IC50) . In this data we can observe a reduction in the number of spheres specifically, indicating the effectivity of the inhibition in targeting CICs. ($^{*}= p < 0.05$).

Supplementary Table 1: Molecular diagnosis of patient gliomas used for experiments of Figure 4E

Sample	Molecular Diagnosis (WHO 2016)
G002	Glioblastoma grade IV IDH1 wild type
G003	Glioblastoma grade IV NOS
G003	Glioblastoma grade IV IDH1 wild type
G004	Anaplastic astrocytoma Grade III NOS
G006	Glioblastoma grade IV IDH1 wild type
